# Leveraging learning management systems in medical education: a scoping review of use, outcomes, and improvement pathways

**DOI:** 10.1080/10872981.2025.2603805

**Published:** 2025-12-16

**Authors:** Sangita G Kamath, Kirtana R Nayak, Veena Nayak, Seemitr Verma

**Affiliations:** a Department of Pharmacology, Kasturba Medical College, Manipal, Manipal Academy of Higher Education, Manipal, Karnataka, India; b Department of Physiology, Kasturba Medical College, Manipal, Manipal Academy of Higher Education, Manipal, Karnataka, India; c Department of Medical Education, Kasturba Medical College, Manipal, Manipal Academy of Higher Education, Manipal, Karnataka, India; d Department of Pathology, Kasturba Medical College, Manipal, Manipal Academy of Higher Education, Manipal, Karnataka, India

**Keywords:** Learning management system, synchronous, asynchronous learning, pedagogy, technology, medical students

## Abstract

Learning Management Systems (LMS) have been increasingly adopted in medical education, serving both undergraduate and postgraduate programs. These platforms are used for various educational purposes, including content delivery, skill development, surgical procedure demonstrations, case-based discussions, and assessments. Despite this, the extent of use and the perceived effectiveness of LMS in medical education remain unclear. Therefore, we planned to conduct a review to explore the usage patterns and the potential benefits of LMS in medical schools. The methodological framework introduced by Arksey and O’Malley was used to conduct this scoping review. Electronic databases PubMed, EMBASE, Web of Science and ProQuest were searched for randomised controlled trials, non-randomised controlled trials (quasi-experimental studies, pretest-posttest designs) and observational studies that considered the effect of LMS in medical schools. The database search and screening process led to the identification of a limited number of studies that met the inclusion criteria and were subsequently included in the qualitative synthesis. The LMS’s used across different medical schools catered to either undergraduate or postgraduate programs. These platforms were employed for a variety of purposes, including content delivery, skill-based teaching, surgical procedure demonstrations, case discussions, and assessments. Student feedback was collected in ten studies, while faculty feedback was reported in only one study. Given that LMS platforms are more likely to benefit students with intrinsic motivation and self-regulated learning behaviors, several studies proposed strategies to enhance LMS utility. These included the use of learning analytics to monitor student engagement and learning patterns, to provide timely feedback and personalised support.

## Introduction

Technology integration into higher education began early, but its adoption accelerated significantly during the COVID-19 pandemic [1,2]. This period marked a substantial shift in learner behaviour, with a growing preference for digital learning and online resources over traditional face-to-face teaching. Later after the pandemic, the trend was highlighted by increased student absenteeism in classrooms with a proliferation of online educational tools. Higher education institutions, including medical schools, sought to streamline technological integration and optimise digital learning in response to these changes. To facilitate these advancements and support e-learning and blended learning approaches, many institutions introduced Learning Management Systems (LMS), fostering innovation in higher education [3]. The adoption of LMS by higher education institutions aligns with the sustainable development goal of ensuring inclusive, equitable, quality education and promoting life-long learning opportunities for all learners [4].

A Learning Management System (LMS) is a web-based technology platform designed to plan, deliver, and manage educational courses [5,6]. It centralises and organises learning resources while enabling seamless interaction between teachers and learners [7]. Faculty can create announcements, structure courses, and provide materials that students can access anytime, anywhere. Students, in turn, can submit assignments through the platform, which allows faculty to grade and provide feedback. Additionally, an LMS can support individualised learning paths, empowering faculty to monitor and track students' progress through features like grade books and analytics.

Integrating technology in higher education, particularly in medical education, significantly enhances the learning experience by offering increased flexibility and opportunities for self-paced learning. Students in competency-based medical education are expected to engage with learner-centred, active, and constructive pedagogical techniques and methods, which lay the foundation for lifelong learning [8]. While the medical curriculum requires learners to attend in-person classroom lectures and interactive discussions, there is also a need for asynchronous contact with students to facilitate a simultaneous independent and collaborative learning experience [7]. LMS has emerged as a scalable solution that provides a comprehensive suite of features designed to support both asynchronous and synchronous learning environments. These systems facilitate various aspects of course development and delivery while accommodating different formats of assessments, thereby guiding the learners to acquire the desired level of competence. The adoption of LMS has enriched traditional teaching methods by encouraging interaction among teachers, students and educational mates [9]. When deployed for announcements and communications, the administrative and management time gets considerably reduced. The students can be guided with assignments so that they spend more time in self and group learning to complete homework and improve their scores.

The adoption and acceptance of technology, particularly LMS in medical education, can be conceptualised using the Unified Theory of Acceptance and Use of Technology (UTAUT) framework [10]. This framework emphasises that educators' belief in the LMS's ability to enhance teaching and learning outcomes which plays a critical role in its adoption. Key determinants include performance expectancy (perceived benefits in teaching-learning outcomes), effort expectancy (ease of use), and facilitating conditions, such as institutional support and training for effective LMS usage. To complement UTAUT, the Delone and McLean Information Systems (IS) Success Model provides a holistic perspective on user satisfaction. It evaluates system quality (reliability, usability), information quality (accuracy, relevance of content), and service quality (support provided) [11]. User satisfaction derived from these factors is a critical measure of the overall success of LMS implementation, offering insights into its long-term viability and impact on educational outcomes. Together, UTAUT explains the factors driving adoption and acceptance, while the Delone and McLean IS model assesses satisfaction and the success of LMS implementation, offering a comprehensive approach to evaluating the benefits of LMS in medical education.

Any new technology, including LMS, requires careful evaluation when introduced into an educational environment. While feedback from learners regarding the content and organisation of educational material has been positive, as well as student-teacher interactions, there are still concerns from both learners and teachers. Students may struggle to take responsibility for their learning and monitor their progress, while educators and administrators face challenges in adoption and implementation due to limited comfort with technology usage by faculty, who are already balancing multiple demands at the university and hospital, including teaching, research, clinical duties, administration, and student mentorship. In light of these concerns, we planned to review the usage patterns and potential benefits of using LMS in medical schools.

## Methodology

In order to conduct this scoping review, we have employed the methodological framework introduced by Arksey and O’Malley [12]. The framework consists of five key stages:

1. **Identifying the research question:** In this paper, we reviewed the use of LMS (concept) in medical schools (context), focusing on undergraduate and postgraduate medical students (population) in accordance with the PCC (population, concept, context) framework to frame the research questions [13]. The research questions identified were 1) How has this technology been utilised to support and supplement teaching and learning activities? 2) How has this platform been used to conduct tests and assessments, providing valuable feedback to learners? 3) What was the feedback of students about the learning management system as they are the primary users of the system along with the faculty?
2. **Locating relevant studies:** The articles were selected from PubMed, EMBASE, Web of Science (WOS) and ProQuest databases (2000 to June 30, 2024) after formulating a search strategy**.** An example of a search strategy used in MEDLINE (via PubMed) is provided in Table 1.
3. **Selecting appropriate studies:** All retrieved records were imported into Rayyan software for management and screening. After automatic and manual duplicate removal, two independent reviewer pairs screened the articles. Reviewers KMP and SV formed one pair, and VN and SK formed the second. Each pair independently screened titles and abstracts against the predefined inclusion and exclusion criteria. Full-text screening was then carried out independently by the same reviewer pairs. Reasons for exclusion at the full-text stage (such as not meeting the population, concept, or context criteria; wrong study design; or insufficient information) were recorded in a screening log. Any disagreements within a reviewer pair were first discussed within the pair. If consensus could not be reached, a reviewer from the other pair acted as an independent adjudicator to resolve the conflict. Inter-reviewer agreement was monitored qualitatively through consistency cheques. In addition, reference lists of included studies were hand-searched (snowballing) to identify any further eligible articles.

**Table 1.** Search strategy.

Population	“medical student*” OR “undergraduate*” OR “postgraduate” OR “graduate medical education” OR internship OR residency OR “undergraduate medical education” OR “postgraduate medical education” OR “medical training” OR “studying medicine” OR “health care training”
	AND
Concept	“learning management system” OR “course management system*” OR “learning content management system” OR “virtual learning environment” OR “integrated learning system*” OR “personal learning environment” OR “blended learning” OR “online learning” OR “computer assisted learning” OR “web based learning” OR “online education” OR “electronic learning management” OR “E-learning tool*” OR “E-learning” OR “virtual simulation”
	AND
Context	“medical school” OR “medical science” OR “health education” OR “medical courses” OR “medical programme*” OR “medicine studies” OR “training of doctors” OR “clinical education” OR “higher education” OR “teaching rounds” OR medical OR education OR “medical education” OR “medicine education” OR “continuing medical education” OR “continuing education” OR “continuing professional development” OR “medical teaching” OR “educational activity”

### Inclusion criteria

- Randomised controlled trials (RCTs), non-randomised controlled trials (quasi-experimental studies, pretest-posttest designs), observational studies studying the effect of learning management systems in medical schools
- Articles restricted to those done in medical schools among undergraduate or post graduate medical students.
- Full-text articles published in the English language were used.

### Exclusion criteria

- Case reports, case series, protocols, systematic reviews and other types of reviews, editorials.
- Articles where the student population is of technical or nursing or allied health sciences background.
- Grey literature - institutional reports, curricula pages, society/college sites, conference proceedings, social media etc.
- Articles published in languages other than English.

4. **Organising the gathered data:** We developed a standardised data extraction sheet informed by the DeLone and McLean Information Systems Success Model, which provides six interrelated dimensions of IS performance: system quality, information quality, service quality, use/usage, user satisfaction, and net benefits. These domains guided the selection of data elements to ensure that the extraction captured both technical and pedagogical aspects of LMS implementation. We extracted data on the following: author, year, country of LMS use, the name of the LMS used, objectives of the study, the population enroled into the LMS, content uploaded, assessments conducted, usage pattern, challenges encountered, advantages of the LMS and plans for improvement. The data extraction sheet (supplementary file I) includes fields mapped to these framework components to ensure consistency and theoretical alignment.

5. **Summarising and reporting the findings**: the relevant findings were organised into thematic categories, with results prioritised according to their relevance to the research questions. The details were extracted and are presented in the Results section below.

6. **Consultation with stakeholders:** This optional sixth stage, consultation, was not undertaken in this review.

We have also adhered to the PRISMA Extension for Scoping Reviews guidelines throughout the process [14].

### Data selection

#### Included articles

**Figure 1.**
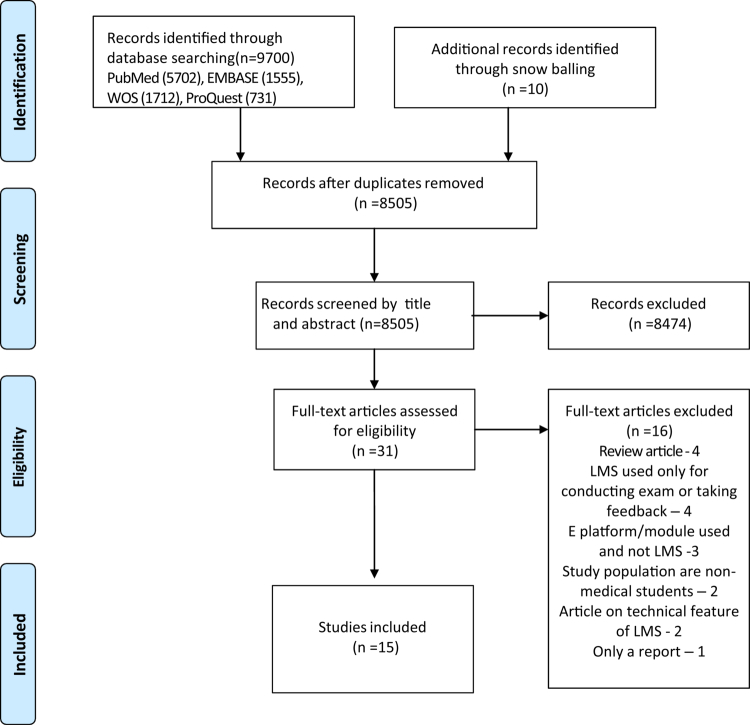
PRISMA flow diagram for the scoping review process. From: Moher D, Liberati A, Tetzlaff J, Altman DG, The PRISMA Group (2009). *P*referred *R*eporting *I*tems for *S*ystematic Reviews and *M*eta- *A*nalyses: The PRISMA Statement. PLoS Med 6(7): e1000097. doi:10.1371/journal.pmed1000097.

The search from the databases yielded 9700 articles. 1195 duplicates were detected and deleted using Rayyan software [15]. The remaining articles were screened for title and abstract out of which only 31 were taken up for full text screening. Among them only 15 were adhering to the selection criteria and were used for qualitative synthesis. The remaining articles were excluded for the following reasons met the exclusion criteria, studies with mixed student population like those from technical, nursing and allied health science background. The flow diagram of the study selection process is given in Figure 1.

#### Data charting and analysis

The final articles for this review were effectively categorised and summarised using seventeen key characteristics we took from literature, and they align with the objectives of this scoping review. These characteristics included authors’ names, year of publication, institution and country, name of the LMS, availability of LMS (free/purchased/developed), aim, population, use of virtual platform projected as LMS, ethics clearance, content added in LMS, assessment methods, features used, feedback from students and faculty, challenges, advantages and improvisation plans. VN created the data extraction sheet and pilot tested it on a small number of studies to ensure it captured all necessary information. VN then shared the data extraction sheet with the team and if any issues arose, they were resolved after discussion. Once the data extraction sheet was compiled, it was cross checked against the original articles by KMP and SK to verify the accuracy of the extracted data.

## Results

**Figure 2.**
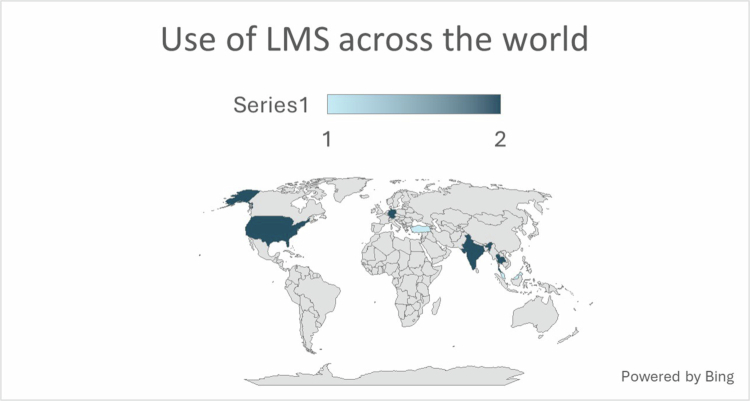
Use of LMS across the world.

The selected articles represented studies conducted in Asia, the United States, Europe, and Australia as shown in Figure 2. The summary of the data of the included studies is provided in Table 2.

**Table 2.** Characteristics of included studies.

Sl. No	Author name	LMS name and the Institute where it was used	Free/purchased/developed	Population	Content added in LMS	Assessment	Pattern of use/features used of LMS
1	Jotwani et al. [16]	Neurosurgery Education and Training School (NETS), All India Institute of medical Sciences, New Delhi, India	Developed By the Department of Neurosurgery, AIIMS, New Delhi and department of Computer Science and Engineering, Indian Institute of Technology, Delhi (IIT-D)	Neurosurgery residents and practitioners all over the globe (free access).	Seminar Presentations of basic neurosurgery Operative Videos, Didactic lectures videos based on procedures, 3-Dimensional (3D) animation based videos addressing basic neurosurgery instruments, patient positions for surgery, Basic and complex surgical approaches Social networking and blogs for addressing queries Tele-education: for neurosurgeons in India and Germany - real classroom interaction in a cost-effective way	Question and answer Forum for open discussion on case based clinical scenarios and discussion of Interesting cases	5226 visitors accessed the platform with 57% of repeat visitors. There were 64,380 views from 190 subscribers. The discussion forum had 968 members from 45 nations. 88.7% of the users used desktops and laptops as their primary tool. About 10% of the visitors used tablets and cell phones to access the contents.
2	Back et al. [7]	Blackboard Academic Suite with the components Learning-, Community- und Content-System (© Blackboard Inc., Washington DC, USA, Charité—Universitätsmedizin Berlin, Germany.	Details of purchase not mentioned. The system was made accessible for all medical students registered at the university	Medical undergraduate students at Charité	Lecture notes or scripts as PowerPoint and PDF. Podcasts Virtual patient cases were provided by the CAMPUS authoring system Discussion forums or quiz formats. “WikiBlog” based on the Team collaboration software Confluence (Atlassian, Australia) and the blogs by the open source web software WordPress MU (Free Software Foundation, USA).	Discussion forums and quizzes	During the semester, 38.6% of all students used the LMS daily, 48.3% on a weekly basis, and 13.1% less than once a week.
3	Johnson et al. [17]	WebCT, Columbus Children’s Hospital (CCH),Ohio State University College of Medicine ⁄Public Health	Details not mentioned	31 postgraduate year 1 (PGY1) residents, 23 PGY2 residents, and 26 PGY3 residents	Ten common diagnoses were chosen for the educational content of Principles of Ambulatory Paediatrics (PAP). Each module had objectives, reading material, a pre-test, and an evaluation. The readings for modules were obtained from journals and textbooks	Pre and post-test were conducted using a software called Respondus	There were 7783 hits to PAP during the 2001–02 academic year. The mean number of hits to the PAP main page for all 80 residents was 95 (SD 56, range 0–222). A total of 1196 hits were made specifically to the readings in the required modules.
4	Halbert et al. [18]	Blackboard, Philadelphia College of Osteopathic Medicine, USA	Details not mentioned	270 1^st^ year medical students enroled in the osteopathic medical school	Along with the course presentations, 29 visual aids and lecture summaries were uploaded. There were 7 brief tables of cytokines and different disease families, as well as 22 hand-drawn, one-page flow charts that summarised the biochemical pathways and bacterial classification methods.	Not mentioned about the assessments conducted	Mean individual student access to the online materials during the course was 6.3 times with a range of 0–42. A total of 82% of enroled students accessed the online learning materials at least once during the course, 66% accessed the online materials two or more times
5	Herbert et al. [19]	Moodle, UNSW Sydney	Details not mentioned	250 students in their second year of Medical Science and Exercise Physiology Programmes at UNSW Sydney.	Recorded lectures and PowerPoints for each module. There were 29 such modules. Each topic concluded with an interactive large group formative assessment session which focussed on the integration of the topic content.	Interactive questions and review quizzes using multiple question types with feedback. The module was not marked complete within the LMS until students obtained a score of 80% or more	74% of enroled students (*n* = 264) had attempted all 29 online modules and 85% of enroled students had completed 90% or more of the modules. The online usage of the modules was determined by data obtained from the SCORM packages
6	Lebeaux et al. [20]	Moodle, Paris Descartes University teaching Board	Available for free	Approximately 800 students of third year of medical curricula	Content was prepared for the infectious diseases and microbiology course. The teaching materials included links to the national syllabus and videos for the topics. Interactive quizzes during the lecture or at the end of the lecture followed by discussion of answers	Continuous assessments were conducted using quizzes	All students made at least one hit with a mean number of 200 ± 146 per student. Highest hits were recorded during the continuous assessment sessions
7	Gaupp et al. [21]	E-learning patient safety course (ELPAS), Freiburg University, Germany	Self created based objectives laid by WHO	340 third year medical students	Video case studies were used. Journal articles, videos, interactive quizzes, and podcasts were used to provide patient safety knowledge	Online quizzes. Reflections on real cases presented to them as a report or short video. Critical peer feedback on their reviews of a scientific paper on patient safety.	193 students participated
8	Chu et al. [22]	Learning moment, Boston university school of medicine	Self developed	Residents of emergency medicine (EM), third and fourth year medical students and physician assistants(42 students)	A platform where students can document and share learning experiences that occur during clinical work.	A three-member faculty panel reviewed the learning moments. The experienced clinical faculty led monthly in-person “Learning Moment Reflection” small group discussions were conducted to discuss and expand upon the learning moments logged during their rotation	Within the first six months after implementation, 42 out of 53 (79.2%) students logged at least one “learning moment” for a total of 323 “learning moments” logged. Students have logged more than 1000 “learning moments”
9	Thepwongsa et al. [23]	KKUMEDX (web-based LMS), Faculty of Medicine at Khon Kaen University, Thailand	Developed by the faculty	283 first-year medical students	20-hour human behaviour course on KKUMEDX, about the human development and behaviour module. In this course, students had twenty 1-h asynchronous online learning sessions and two 1-h sessions for class discussion with the course lecturers. The total number of videos were 77 and the length of each video ranged from 0.22-49.01 min. The total time of the videos for each topic ranged from 8.48-70.33 min.	Multiple-choice test was conducted on Google Forms. Interviews were conducted online, in and recorded through Google Meet, together with a writing record by the interviewer (IT). All audio recordings were thematically analysed.	The total learning time of the students ranged from 0-6848 min, and a mean of 908.13 (SD 933.24) minutes. In terms of students' learning behaviour, 134 (47.35%) students spent _10 h on the course and they often logged in to the course during the morning (2780 logins, 45.3%) and evening hours (1678 logins, 27.3%). There was no association between the good score and the course attendance.
10	Seluakumaran et al. [24]	Moodle integrated a Moodle e-learning site called DPhysiol, Department of Physiology, Faculty of Medicine, University of Malaya, Kuala Lumpur, Malaysia	we designed the Moodle site to include features that would promote active learning and enable uploading of relevant course information and lesson materials	All first-year MBBS students (95 men and 121 women)	DPhysiol content was designed using the following topic outlines: Interactive session, course information, lecture notes, laboratory exercise, problem solving session, AV resources, quizzes, useful links	*Online activity logs* *Exam scores* For the purpose of this study, the physiology component marks in *part A* and *part B* exams were compared with the activity logs of individual students obtained from DPhysiol during *terms 1* and *2*, respectively. To assess the Moodle usage between high and low achievers, we also compared the activity logs of students who were in the top 10% of the overall final physiology results (*part A* and *part B* exams combined) with those of students who were in the bottom 10%. We also assessed student participation and performance in online quizzes and correlated these with their final exam marks Finally, to evaluate the value of Moodle in improving student exam performance, mean final physiology marks obtained by the students (academic session 2008/2009) were compared with marks obtained by the previous class (academic session 2007/2008), which did not use the CMS	About 90% (*n* _ 194, 86 men and 108 women) of first-year MBBS students registered as DPhysiol users. A total of 178 students (72 men and 106 women) participated. Of the registered DPhysiol users, 90.7% of the students (*n* _ 176) enroled within the first 2 weeks. The site recorded a total of 6,347 visits and 51,935 hits during the entire academic session The mean number of hits on weekdays (204 _ 160 hits/day) was significantly higher than that of weekends (141 _ 108 hits/day, *P* _ 0.05) during the study period. The usage gender-wise was 0.98 _ 0.81) and 0.84 _ 0.65 for male and female students, respectively. The most frequently used content was the discussion forum (5,979 hits), announcement forum (2,766 hits), and chat function (122 hits). Other frequently used resources were lecture notes (8,075 hits), quizzes (4,006 hits), and AV resources (2,580 hits). The average access per uploaded lecture note was 192 hits/file, whereas accesses for AV resources were 107 hits/file. The discussion forum had a total of 54 posts (52 by students and 2 by tutors) and 121 replies (105 from students and 16 from tutors). For the announcement forum, there were 35 postings by tutors.
11	Kolcu et al. [2]	**Moodle,** Suleyman Demirel University Faculty of Medicine, Isparta, Turkey	The rights of the LMS are given to the faculty administration, and its management is left to the Department of Medical Education and Informatic	1645 students of Süleyman Demirel University, School of Medicine (SDUSM)	Course videos, learning resources, Debate forums for students were provided, homework and task management were made.	1023 learning sources, 589 live lectures were made and total 345 hours of video records taken between a total of 40 measurement/ evaluation applications including 37 shelf exams, 5 internship exams, and 3 preclinical board exams within one month.	-
12	Thepwongsa et al. [25]	KKUMEDX, Faculty of Medicine at Khon Kaen University, Thailand	Developed by faculty	Khon Kaen University learners	Content presentation only (e.g., text only, audio lectures with slides, and text with multimedia materials); interaction with content (e.g., cases with questions, quizzes, assignments); and interpersonal interaction (e.g., discussion forum, chat, e-mail).	A question bank was created with MCQs Quizzes were also conducted	
13	Dash [26]	**Google classroom** free of cost with G-suite, **MMMC, Manipal**	Not mentioned	83 first year MBBS students	Lecture notes, which included the PowerPoint presentation to be used in the lectures and incomplete notes for class activities; a quiz with multiple-choice questions, short answer questions, and multiple-true-false questions; learning resources, such as links to articles and YouTube videos that explained concepts; notifications about upcoming classroom activities; and assignments that needed to be turned in via the Google Classroom platform were among the contents.	Assessment details not mentioned	The details on usage was not mentioned however 41 students were enroled
14	Çakmakkaya et al. [27]	CANVAS Learning Management System, Instructure, Salt Lake City, UT, USA, Cerrahpaşa Medical Faculty, Istanbul	**Commercial**	All Cerrahpaşa Medical Faculty students studying online in the 2020 academic year spring semester.	An announcement page, a content page including previously recorded lectures, slideshows, and learning resources, an assignment and exam page, and an overall course page were all part of the system. Both synchronous live streaming and recording of lectures were used to offer asynchronous learning possibilities. The presentations and preparatory materials were available to students in advance.	Details on assessments were not mentioned	1050 students were enroled. 47% students used laptops,42% phones, 6% tablets 3% desktops to access the content. Most students (60%) reported the focus time on online lectures as 20–40 min
15	Gokli et al. [28]	Absorb; Calgary, Alberta, Canada RADIAL (Radiology’s Intelligent Adaptive Learning), Department of Radiology, Staten Island University Hospital. 475 Seaview Ave., Staten Island, NY 10305, USA	**Commercial** In terms of ongoing costs, the interface for the LMS costs $1,800 per month	Paediatric radiology dept	Each of the 289 courses in the LMS, which were divided into 18 subtopics, included reference articles, multiple-choice quizzes, and recorded lectures. A toolkit tile that includes department-produced PDF tutorials, highly cited articles, 15-minute short lectures, figures and tables from important papers or lectures, and report templates. Also the LMS had a ultrasound checklist app which can be used by trainees.	MCQs and quizzes were conducted for formative and summative assessments. Qualitative surveys, review of course comments and focus groups were used for assessments	There were 853 enrolments, 39 new users, 2702 logins by learners over 30 days, and the LMS had 46 courses.

**LMS platforms:** Several medical schools utilised LMS platforms developed in-house, tailored to their specific educational needs. Examples include the ‘Neurosurgery Education and Training School (NETS)’, a collaborative initiative by the Department of Neurosurgery at AIIMS, New Delhi, and the Department of Computer Science and Engineering at the Indian Institute of Technology, Delhi (IITD); the ‘E-learning Patient Safety Course (ELPAS)’ developed by Freiburg University, Germany; ‘Learning Moment’ by Boston University School of Medicine; ‘KKUMEDX’, a web-based LMS developed by the Faculty of Medicine at Khon Kaen University, Thailand; and ‘RADIAL’ (Radiology’s Intelligent Adaptive Learning) created by the Department of Radiology at Staten Island University Hospital, USA.

**Figure 3.**
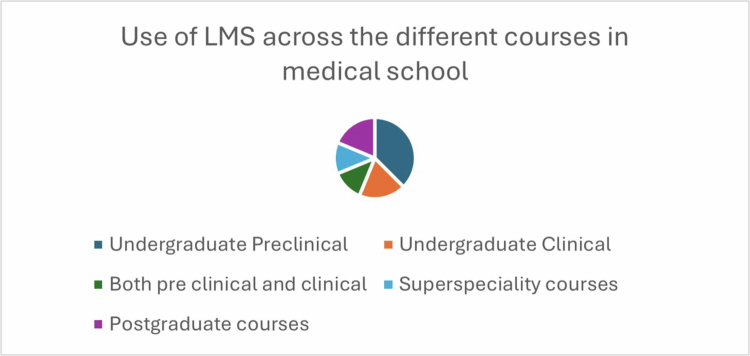
Use of LMS across the different courses in medical school.

Other medical schools employed commercially available or freely accessible LMS platforms such as the Blackboard Academic Suite—which includes Learning, Community, and Content systems (© Blackboard Inc., Washington DC, USA)—as well as WebCT, Moodle, Google Classroom, and the CANVAS Learning Management System. The LMS was used in the medical schools across the professional years of training as shown in Figure 3.

## Content uploaded on the LMS

**Figure 4.**
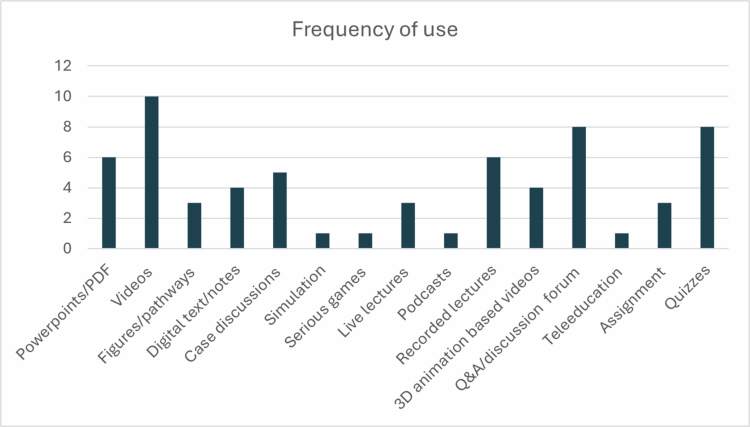
Frequency of uploaded content on the LMS.

A wide variety of educational materials were uploaded to the LMS platforms to enhance learning and engagement as shown in Figure 4. These included:

- Seminar presentations in the form of PowerPoint slides or PDFs
- Videos demonstrating surgical procedures and skill-based training
- Asynchronous and live-streamed didactic lectures
- 3D animation-based educational videos
- Lecture notes supplemented with flowcharts, including some hand-drawn diagrams, biochemical pathways provided as scripts or PDF documents
- Podcasts and audio-based learning materials
- Virtual patient cases and case-based discussion modules
- Journal article reviews and discussions
- Laboratory exercises and lecture notes offered as pre-reading material
- Problem-solving cases and curated links for extended learning

Interactive elements, such as quizzes and multiple-choice questions, were integrated into some lectures to promote active learning and maintain student interest. In addition, many platforms featured social networking tools, blogs, and discussion forums to encourage peer interaction, resolve student queries, and facilitate academic debates. Assignment submission tools were also utilised to manage coursework and conduct self-directed learning assignments effectively.

Some institutions extended the use of their LMS to support tele-education initiatives, enabling collaboration and content sharing across different academic institutions.

## Assessments conducted on LMS

Continuous and formative assessments were integrated into the LMS across several institutions. Some employed interactive case-based question-and-answer discussions to engage learners actively.

The most used assessment formats included quizzes, as well as pre- and post-tests accompanying lectures or instructional videos. These were typically presented as multiple-choice or true/false questions. In addition to objective assessments, reflective writing based on presented cases was also utilised to encourage deeper understanding and critical thinking. Selukumaran et al. reported the use of paper-based submissions for assessments following LMS-based activities, blending traditional and digital approaches to evaluation [24].

## Advantages of the LMS

Articles have highlighted several key advantages of using LMS, with ease of use and user-friendly interfaces being the most frequently cited [7,22,25]. LMS platforms allow for easy updating of content, enabling the integration of modern technologies, procedures, and skills into the curriculum. They support self-paced learning, which enhances flexibility and convenience for students [16,26].

The ability to provide immediate feedback following assessments fosters deeper engagement and motivates students to improve their performance. Additionally, LMS tools facilitate the documentation of student participation and academic progress [17].

A wide variety of interactive teaching strategies can be implemented through LMS platforms, contributing to a more engaging learning experience. Many systems also support peer and cooperative learning through discussion forums, helping bridge the gap between learners and between students and educators [20].

This is particularly valuable in demanding programmes such as medicine, where faculty and students often have limited time for real-time interaction. The asynchronous nature of LMS-based learning allows students to engage with content and participate in discussions at their convenience [24,26]. Furthermore, the use of freely available platforms such as Google Classroom has increased accessibility and acceptance among students [26].

**Challenges with the use of LMS:** From the students’ perspective, the use of LMS presents relatively few barriers. However, students have suggested that certain modifications, particularly in assessment patterns, could improve the experience. One study reported that students found the interactive features of LMS less engaging, primarily due to the absence of incentives. Additionally, some students expressed dissatisfaction with lecture notes being uploaded only after the class sessions and found it better if uploaded before the class [24].

Technical issues also pose challenges. Poor Wi-Fi connectivity across campuses can hinder access to lecture materials, and some students reported difficulties uploading assignments via handheld devices [26]. Another significant concern is the limited ability of LMS platforms to effectively assess the attitudes and practical skills of medical students [26]. There are also concerns about the use of LMS for high-stakes (summative) assessments, as the platforms may lack the necessary validity for such purposes [20]. While LMS tools can be highly beneficial for self-directed learners, they may not be ideal for students who struggle with self-regulation and independent study habits.

In some institutions, especially in developing countries, broader challenges hinder LMS adoption. These include inadequate infrastructure, limited bandwidth, and a lack of administrative support. As a result, institutions may need to rely on lighter versions of LMS platforms that demand fewer resources (16,19) Furthermore, the need to validate content before uploading it often goes unmet due to time constraints, resulting in the dissemination of unverified material [16].

Financial investment is another substantial barrier; robust LMS platforms often require significant funding [16]. Faculty members also face considerable demands, as they must create, validate, and structure content to be interactive and pedagogically sound [7,17]. This process is time-consuming and may detract from their research commitments [17].

## Plans to improve the usage of LMS

1. **Integration of Self-Evaluation Modules**: Introduce self-assessment tools such as Computer-Based Tests (CBT) and Internet-Based Tests (iBT) to promote active learning and allow students to monitor their progress independently [16].
2. **Provision of Synchronous Learning Opportunities**: Enhance live learning through webinars, real-time assignments, and online certification programmes. Ensure these are freely accessible to all trainees globally, fostering inclusivity and international collaboration [16].
3. **Incorporation of Innovative Features to Enhance Engagement**: Implement novel teaching tools and interactive elements designed to capture and sustain student attention, such as gamification, virtual simulations, or case-based discussions [7].
4. **Adoption of Proven Educational Tools**: Utilise evidence-based digital tools that have demonstrated potential benefits in medical education, ensuring alignment with the specific needs of faculty and learners aiming for high-quality instruction [7].
5. **Retention of Access to Previous Learning Materials**: Maintain student access to earlier course content and resources to facilitate revision, longitudinal learning, and integration of knowledge [7].
6. **Continuous Monitoring of Learner Behaviour**: Implement analytics to observe and assess student interaction with LMS materials, allowing for timely interventions and tailored support [25].
7. **Enhanced Data Analytics and User-Friendly Features**: Provide comprehensive usage data, including viewing history, log files, and interaction patterns. Improve usability through features like easier image uploads, advanced Microsoft Word processing tools, and a streamlined interface [26].
8. **Responsive Content Delivery**: Adjust the length and format of lectures based on attention span research. Address resource disparities by supporting students with technical and workplace-related challenges, ensuring equitable access to learning [27].

**Feedback from faculty:** Faculty input was only collected for one research, and most professors thought that Moodle aided in instruction and intended to keep using it in the upcoming academic years [20].

**Feedback from students:** Feedback from students was reported in ten articles. Overall, students expressed great satisfaction with the online courses that were available on the learning management systems. Most students (70.7%) used the lecture slides, with videos (71.9%) and digital texts (71.3%) coming in second and third, respectively. Discussion boards (7%), serious games (13.8%), and simulations (33.4%) were the least popular tools. Students utilised LMS to obtain study materials (68.3%), to prepare for tests (63.3%), and to prepare and improve their learning (54.5%). 2.2% of students utilised the LMS to communicate with their peers, 1.9% to communicate with teachers, and 1.3% to keep track of lists and calendars [7]. The residents in the Johnson CE et al. study concurred that the information had applications to patient care and that each module component was advantageous [17]. According to Halbert et al., 90% of respondents said that the online course improved their ability to prepare for it. Of the students who used the online resources, 57% said that they ‘rarely’ or ‘never’ substituted the course materials [18]. After utilising the online courses, students in another study by Herbert C. et al. self-reported a notable improvement in their comprehension of the subjects. Students reported that the modules helped them determine their overall study strategy and the areas in which they most needed to concentrate their efforts [19]. In order to lower the possibility of cheating, students also recommended cutting down on the amount of time allotted for answering questions and increasing the amount of time for feedback. Additionally, they asked for a week to pass between a lecture and the associated question [20].

Most participants in the study that utilised Dphysiol LMS gave positive feedback (strongly agree/agree) about the simplicity of use (78.2%), accessibility (67.8%), and registration process convenience (71.8%). The majority of users (70.1%) expressed satisfaction and happiness with DPhysiol. 88.5% of respondents felt that the materials were helpful, and 85.1% of respondents thought the resources in DPhysiol were appropriate and relevant. Regarding the usage of DPhysiol as a communication tool, 55.1% of respondents agreed that it facilitated student-lecturer engagement, while 56.9% of respondents thought it allowed interactions with other students. Regarding the topic of whether DPhysiol could take the place of traditional learning, the majority of respondents had neutral (31.4%) to negative (30.7%) opinions. While acknowledging that DPhysiol enhanced conventional in-person instruction, (59.8%), students still preferred to learn through conventional lessons. Respondents (78.1%) strongly recommended the continuation of DPhysiol and 82.2% of them proposed that a similar e-learning tool be used by other departments in the medical faculty (16) In the study by Kolcu G et al., where LMSAS LMS was used, student feedback was obtained in four subdomains [2]. These included performance expectancy (PE), which reflects the expectation of improved performance with technology use; effort expectancy (EE), representing the belief that the technology will be easy to use; facilitating conditions (FC), indicating the presence of supportive elements; and social influence (SI), referring to the opinions of important people in the environment where the technology is implemented. In the analysis of scale scores for all students, the mean scores were as follows: performance expectancy—22.70 ± 9.48, effort expectancy—15.50 ± 5.79, facilitating conditions—14.92 ± 5.61, social influence—6.70 ± 3.37, and the total score—59.84 ± 21.61. In comparing the preclinical and clinical years, it was seen that there was a statistically significant difference between the preclinical students and the clinical students in both total scores and all sub-dimensions [2]. In the study conducted by Dash et al., 80.5% of students reported that accessing class notes via Google Classroom was easy, and the same proportion found the YouTube videos shared on the platform helpful. Meanwhile, 17.1% of students remained neutral about its usefulness. Although 68.3% found it easy to answer quizzes on Google Classroom, 12.2% were neutral, and 7.3% found it difficult [27]. According to Çakmakkaya OS et al., students' overall satisfaction with the LMS was positively correlated with their familiarity with online teaching techniques, good IT literacy, satisfaction with faculty members' online teaching abilities, faculty members' interactive teaching methods, and self-reported longer attention span during online learning. Technical difficulties were negatively correlated with students' overall satisfaction. They opined that seminars in lecture halls are more productive [27].

## Discussion

The Learning Management System (LMS) has become an essential component of the learning environment in higher education, particularly in medical education. It is designed to supplement teaching, learning, and assessment by integrating multiple functions, such as course delivery, communication, and evaluation, into a unified digital platform. The LMS supports both individual and collaborative learning, enabling a wide range of interactions including student-to-student, student-led, and teacher-student communication. LMS facilitates the administration and management of the learning process by supporting the instructors in learning design and creating more engaging courses, enabling effective learner participation, access, and reuse of digital learning materials through a single platform.

An ideal Learning Management System (LMS) should support the upload and download of electronic documents, spreadsheets, presentations, images, animations, and audiovisual content. It should facilitate a range of assessments, diagnostic, formative, summative, and self-assessments, to evaluate and monitor student performance. Additionally, it should enhance student–faculty interaction through both synchronous and asynchronous communication tools, while enabling academic administrators to efficiently monitor and manage classes and learning materials [29]. According to the DeLone and McLean Information Systems Success Model, user satisfaction is influenced by the accuracy, relevance, and timeliness of the course content provided [11]. Course educators and faculty enhance LMS utility by offering curated resources such as multimedia presentations, videos, podcasts, recorded lectures, lecture notes, virtual patient cases, and pre-class [7,16,17]. A study by Herbert C et al. reported that 82% of enroled students accessed course materials at least once during the course, with 66% returning for multiple visits [19].

### LMS is not just a content repository

LMS usage extends beyond passive content consumption. It actively engages learners through formative and continuous assessments, including quizzes, assignments, and reflective writing tasks typically supported by timely faculty feedback [19,21,22]. LMS platforms also facilitate asynchronous and synchronous discussion forums, fostering meaningful engagement between faculty and students [16,18,26]. This is particularly valuable in time-constrained medical curricula, where physical classroom and hospital-based learning opportunities are limited.

Although LMS utilisation patterns may vary depending on the course type and student level (undergraduate vs postgraduate), research indicates a positive correlation between participation in LMS-based assignments and final examination performance [24]. While online quizzes remain the most commonly used assessment modality, the evaluation of reflective writing has also been employed to foster critical thinking and encourage reflective practice [21,22]. The LMS effectively supports formative and continuous assessments, enabling faculty to design, launch, and evaluate assignments, while allowing students to submit their work and monitor their scores and academic progress. Feedback on assignments and assessments, beyond just scores and grades, plays a crucial role in helping learners improve their performance. LMS platforms support this through the provision of descriptive feedback aligned with scoring rubrics. Additionally, as reported by Gaupp R 2018 et al, critical peer feedback serves as an effective strategy to promote peer-to-peer learning among medical students [21].

Even in higher education settings, students primarily engage with LMS platforms to access supplementary materials for further reading [6]. However, our synthesis highlights the potential for LMS to evolve beyond content delivery toward more integrated functions, including open curriculum design, e-portfolio management, structured online teaching, and learning assistance. This perspective extends previous research by emphasising the transformative role of LMS in supporting longitudinal learning, reflective practice, and personalised learner paths.

### Effort expectancy and LMS

A key factor influencing the successful implementation and adoption of educational technologies like LMS is ease of use, which also significantly affects user satisfaction [10]. Most studies report that a user-friendly interface and platform adaptability by both students and faculty contributes to its effective utilisation. The early upload of content and the provision of easily accessible learning materials support self-paced, flexible learning, aligning well with the diverse needs of learners in medical education. Successful technologies are those that make teaching and learning easier and more productive, and the LMS supports this by facilitating both processes effectively. However, additional dimensions, such as interactive learning design, feedback and analytics dashboards, and competency tracking systems that enhance student engagement and satisfaction, can further strengthen its educational impact and sustainability (smart cities).

### LMS for performance expectancy

According to student feedback on performance expectancy, as outlined in the Unified Theory of Acceptance and Use of Technology (UTAUT) model, learners self-reported a notable improvement in their comprehension of subject matter. Students indicated that the LMS modules aided in shaping their overall study strategies and helped them identify areas requiring greater focus and effort. Not many studies other than Seluakumaran et al attempted to explore the correlation between usage of LMS and examination performance and they found the pattern of use between top 10% and bottom 10% of students were no different. Although, students acknowledged that LMS-based tasks enhanced conventional in-person instruction [19] the majority still expressed a preference for traditional classroom learning, reinforcing the view that LMS serves best as a supplement for face-to-face teaching in medical education. Research on blended learning in higher education similarly indicates that the LMS functions primarily as a supplementary tool and cannot replace the pedagogical role of teachers or the value of direct, face-to-face communication [6]. Nevertheless, LMS platforms must continue to evolve, to strengthen the reliability of assessments used to evaluate student success, automate reward schedules, and enable the monitoring of programme performance to identify areas for improvement. Such enhancements could elevate the LMS from a supplementary tool to a more integrated component of the learning ecosystem, thereby expanding its impact on educational outcomes.

### Challenges with LMS

One of the key challenges in the adoption of LMS by students is its limited ability to improve performance among those who lack self-directedness, self-regulation, and independent study habits. These students often engage inconsistently with LMS resources and tend to focus on less demanding tasks, which limits the platform’s educational potential. This highlights the need for additional support and structured scaffolding to help cultivate autonomous learning behaviours. The incorporation of interactive elements, such as gamification, has been suggested as a strategy to enhance student engagement with LMS platforms [7]. The faculty often face challenges in creating curated, pedagogically sound resources and ensuring their timely upload. Students have expressed a clear preference for course materials to be made available before class sessions rather than after, as it supports better preparation and active participation [20].

### LMS for measuring progress of learning

An LMS serves as a strategic tool for educational institutions, with one of its greatest advantages being the ability to provide deeper analysis and insights into the learning process. A well-designed LMS fosters a learner-centred approach by supporting the institution not only in learning, but also in tracking progress and measuring achievement [30]. A few studies have reported the use of online activity logs and the number of content “hits” to track LMS usage by learners [3,20]. Enhanced data analytics, such as comprehensive usage data including viewing history, log files, and interaction patterns, can provide valuable insights for administrators and educators. Learning analytics obtained allow education organisations to continuously improve their learning process efficiency. This data can inform decisions related to LMS adoption, content improvement, and the planning of faculty development programmes aimed at strengthening digital teaching competencies. In addition, robust technical support on campus is essential to troubleshoot login and access-related issues, thereby ensuring equitable and uninterrupted access to the LMS for all stakeholders. This is particularly important, as technical difficulties have been shown to correlate negatively with overall user satisfaction and can hinder effective engagement with the platform.

### Limitations and future directions

The scope of this review was limited to studies involving medical students at both undergraduate and postgraduate levels, excluding those in allied health or technical programmes. Consequently, the findings may not apply to educational settings outside medical schools, as learning needs and LMS usage may differ in other disciplines. This review also excluded research where the LMS served solely as a platform for administering questionnaires related to LMS usability or for isolated assessments, as these uses did not align with the review’s focus on broader, sustained integration of LMS in medical curricula. As a result, the synthesised evidence primarily reflects the structures and experiences of medical education institutions, and caution should be exercised in generalising the conclusions beyond that context. A limitation of this review is that grey literature sources (such as conference abstracts, theses, and reports) and articles published in languages other than English were not included, which may have led to omission of relevant evidence not published in peer-reviewed journals.

## Conclusion

The present scoping review indicates that learning management systems are widely implemented in medical education for delivering course materials, facilitating exam preparation, and supporting efficient organisation of teaching and learning activities. Medical students appreciate the accessibility and clarity provided by LMS platforms, and value their integration with interactive and face-to-face learning experiences. Performance expectations, ease of use, and availability of support services further shape student satisfaction and engagement with LMS tools in both undergraduate and postgraduate programmes.

Future research can focus on exploring the relationship between LMS usage and the achievement of learning outcomes, including the development of soft skills such as communication and procedural competencies. Additionally, studies can investigate the impact of faculty development programmes on enhancing the adoption, effective utilisation, and pedagogical integration of LMS in medical education. Learning analytics (LA) involves analysing educational data to enhance the learning experience, for example, by monitoring the time a student spends on the course or the frequency of their visits. Future research can explore how institutions harness LA from LMS platforms to track student progress and empower both educators and learners to make well-informed, data-driven decisions. The LA functionalities embedded within the LMS can enable educational institutions to continuously improve the learning and assessment processes.

## Supplementary Material

Supplementary material Supplementary file PRISMA checklist

Supplementary material supplementary file Compiled data extraction sheet

## Data Availability

Data will be shared upon reasonable request.
