# Supplementary material for: Leveraging learning management systems in medical education: a scoping review of use, outcomes, and improvement pathways
Source: Med Educ Online. 2025 Dec 16;30(1):2603805. doi: 10.1080/10872981.2025.2603805 (PMC12713214; doi:10.1080/10872981.2025.2603805)
Supplement: Supplementary material — supplementary file Compiled data extraction sheet [file ZMEO_A_2603805_SM7350.docx]

| **Sl.No** | **Author name** | **Year** | **Institute, Country** | **LMS name** | **Free/Purchased/Developed** | **Objectives of the study** | **Population**  **(For whom, how many, course)** | **Ethics clearance** | **Use of virtual platform projected as LMS** |
| --- | --- | --- | --- | --- | --- | --- | --- | --- | --- |
| 1 | Jotwani P et. al | 2014 | All India Institute of medical Sciences , New Delhi, India | Neurosurgery Education and Training School (NETS) | Developed  By the Department of Neurosurgery, AIIMS, New Delhi’ and department of Computer Science and Engineering, Indian Institute of Technology, Delhi (IIT‑D) | - development and utility of multi-authored, interactive, global, free‑access, open source e‑learning platform in neurosurgery with the integration of web-based technologies and tele‑education - effectively use technology to change neurosurgical education from a traditional apprenticeship based summative system to a formative evaluation based system with incorporation of skills training. | neurosurgery residents and practitioners  free access for practitioners all over the globe.  Over a period of one year they had 5226 visitors with 57% of repeat visitors;  425 visitors/month from 73 countries | Not mentioned | No |
| 2 | Back A et al | 2016 | Charité – Universitätsmedizin Berlin, Germany. | Blackboard Academic Suite with the components Learning-, Community- und Content-System (© Blackboard Inc., Washington DC, USA) | Details of purchase not mentioned  The system was made accessible for all medical students registered at the university | To evaluate the medical students’ needs and expectations concerning e-learning tools and learning management systems, based on their current use. | Medical undergraduate students at Charité | Obtained | No |
| 3 | Johnson CE et al | 2004 | Columbus Children’s Hospital (CCH),Ohio State University College of Medicine ⁄Public Health | WebCT | Details not mentioned | To describe the WebCT course, Principles of Ambulatory Paediatrics which was designed for paediatric residents during a month long rotation known as the ambulatory block month (ABM) | 31 postgraduateyear 1 (PGY1) residents, 23 PGY2 residents, and 26 PGY3 residents completed the ABM | This course of Paediatrics was a mandatory course for paediatric residents and was thus exempt from Institutional Review Board . However there was a disclaimer each time the residents logged on to the course that would be collected anonymously.  Thereby logging-on to the course by residents granted consent to use data from that resident. If residents did not log-on to the course, they still had access to paper copies of on-line readings. | No |
| 4 | Halbert C | 2011 | Philadelphia College of Osteopathic Medicine, USA | Blackboard | Details not mentioned | 1. To quantify the use of online learning materials within a preclinical course delivered to first-year osteopathic medical students; 2. To determine the perceived benefit of these materials; and 3. To determine if students with particular learning styles preferentially utilized online learning materials | 270 1^st^ year medical students enrolled in the osteopathic medical school | Obtained | No |
| 5 | Herbert C | 2017 | UNSW Sydney | Moodle | Details not mentioned | 1. To evaluate the approach of blended learning in terms of student utilisation of online modules and interactive large-group teaching sessions as well as student perception of the role of online resources for their learning | 250 students in their second year of Medical Science and Exercise Physiology Programs at UNSW Sydney. | Obtained | No |
| 6 | Lebeaux D et al | 2021 | Paris Descartes University teaching Board | Moodle | Available for free | To assess the effects on student attendance, satisfaction and performance of introducing Moodle as a teaching interface | Approximately 800 students of third year of medical curricula | This project was approved by the Paris Descartes University teaching board. Only oral consent was taken from the students |  |
| 7 | Gaupp R | 2018 | Freiburg University, Germany | E-learning patient safety course (ELPAS ) | Self created based objectives laid by HWO | - To know which factors were relevant to e-learning acceptance by medical students. - To know whether the perceived usability of the e-learning system would influence evaluation results. | 340 third year medical students | Obtained |  |
| 8 | Chu A et al | 2019 | Boston university school of medicine | Learning moment | Self developed | To understand the importance of a learner-centered model of instructional design  To evaluate the usability of LM and identify features that enhance adoption by users. | Residents of emergency medicine (EM), third and fourth year medical students and physician assistants(42 students ) | Exempted from review | System Usability Scale (SUS) questionnaire |
| 9 | Thepwongsa I et al | **2021** | Faculty of Medicine  at Khon Kaen University, Thailand | KKUMEDX (web-based LMS) | **Developed by the faculty** | Assess the effects of the newly developed  LMS on the students' usage, satisfaction, and learning outcomes;  and identify medical students' perceptions, and factors influencing their  adoption of this LMS | 283 first-year medical students  In total, 157 out of 283 (55.5%) first-year medical students participated  in the questionnaires of this study  Among them, 15 students were sought due  to the convenience of the online semi-structured interviews. Five students  from each of the following groups were recruited: regular attendance  in the course; partial attendance; and rarely or never attended the  online course for study. | This study was provided by the Khon Kaen University Human  Research Ethics Committee (Project number HE631031). | **No** |
| **10** | **Seluakumaran K et al** | **2011** | *Department of Physiology, Faculty of Medicine, University of Malaya, Kuala Lumpur, Malaysia* | Moodle  integrated a Moodle e-learning site called  DPhysiol into the existing face-to-face teaching of physiology  to first-year undergraduate medical students. | we designed the Moodle site to include features that would  promote active learning and enable uploading of relevant  course information and lesson materials | To evaluate the usage pattern of Moodle and its impact on student learning outcomes using online log activity, student  exam performance, and student feedback from questionnaires. | At the beginning of *term 1*, all first-year MBBS students (95 men and 121 women, age: 20.10 _ 0.49 yr, mean _ SD) were requested to self-register at the DPhysiol site using individual user names and e-mail addresses. They were given a short briefing about DPhysiol during their introductory week (all of them were first-time Moodle  users). Along with a set of instructions on the registration, students were also provided with an enrollment key, which acted as an access code to the site. When the enrollment key was first entered, students  were automatically placed into 10 separate groups based on their  tutorial grouping assigned by the faculty. Enrolment to DPhysiol was made available from the beginning of *term 1* until the end of *term 2* and was not made compulsory. | **Not mentioned** | **no** |
| **11** | **Kolcu G et al** | **2020** | Suleyman Demirel University Faculty of Medicine, Isparta, Turkey | **Moodle** | The  rights of the LMS are given to the faculty administration,  and its management is left to the Department of  Medical Education and Informatic | to evaluate the students’ acceptance  and satisfaction about the learning management  system during the transition to distance education due  to the COVID-19 pandemic in Süleyman Demirel  University Faculty of Medicine’s Medical Education  Program. | The study population was determined as the  students of Süleyman Demirel University, School of  Medicine (SDUSM) (N: 1645). The scale was sent to  all students online, and 941 students reported opinions  (n: 941) (access rate 57.20%). | For this study, approval was obtained  from the Süleyman Demirel University, School of  Medicine, Ethical Committee of Clinical Research. | **no** |
| 12 | Thepwongsa I et al | **2023** | Faculty of Medicine  at Khon Kaen University, Thailand | KKUMEDX | **Developed by faculty** | The present study examined learners’ experiences with the online LMS. | This study was conducted with Khon Kaen University learners registered and enrolled in online courses through KKUMEDX. There were no exclusion criteria in this study. | This study was approved by the Human Research Ethics Committee of Khon Kaen University (project number HE631031). | **no** |
| **13** | **Dash S** | **2019** | **MMMC, Manipal** | **Google classroom**  free of cost with G-suite, | **Not mentioned** | Google Classroom as a Learning Management System for teaching and learning a module of biochemistry and to  assess its efficacy and acceptability among first year MBBS  students of Melaka Manipal Medical College. | **83 first year MBBS students** | **Not mentioned** | **No** |
| 14 | Çakmakkaya 0S et al | **2024** | Cerrahpaşa Medical Faculty, Istanbul | CANVAS Learning Management System, Instruc­ture, Salt Lake City, UT, USA | **commercial** | for assessing stu­dents’ overall satisfaction with Emergency Remote Teaching and for defining the main factors that contrib­ute to students’ satisfaction in the online environment at Cerrahpaşa Medical Faculty.  we aimed to understand what kind of challenges students faced dur­ing online learning. | target population was all Cerrahpaşa Medical Faculty students studying online in the 2020 aca­demic year spring semester. | The study was designed and conducted in accordance with the principles of the Declaration of Helsinki. Ethics Committee of the Cerrahpaşa Medical Faculty approved the study (Number: 13549728-619-169921). | **no** |
| 15 | Gokli A et al | **2021** | Department of Radiology, Staten Island University Hospital,  475 Seaview Ave., Staten Island, NY 10305, USA | Absorb; Calgary, Alberta, Canada  the pediatric radiology LMS was launched  as RADIAL (Radiology’s Intelligent Adaptive Learning).  This innovative LMS is connected to our institutional picture  archiving and communication system (PACS) for ease of navigation | **Commercial**  In terms of ongoing costs,  the interface for the LMS costs $1,800 per month | aim of this  paper is to describe LMS development for pediatric radiology in  a way that is scalable for implementation outside our institution | **Pediatric radiology dept** | **No mentioned** | **no** |

| **Sl.No** | **Author name** | **Content added in LMS** | **Assessment** | **Pattern of use/features used of LMS** | **Feedback from faculty** | **Feedback from students** | **Challenges** | **Advantages** | **Improvisation plans** | **Miscellaneous** |
| --- | --- | --- | --- | --- | --- | --- | --- | --- | --- | --- |
| 1 | Jotwani P et. al | 1. Presentations: 344 seminars as ppt and.pdf for basic neurosurgery discipline and allied sub specialties (vascular, skull‑base, pediatrics, neuro‑oncology, functional, spine, peripheral nerve, neuro‑trauma), and recent advances in the field 2. Videos: 26 edited operative videos describing the standard microscopic and endoscopic procedures in neurosurgery for various pathologies illustrating the surgical steps 3. Didactic lectures videos: 37 videos addressing various task based and procedure based sessions (addressing microsuturing skills training, neuroendoscopic procedures and high speed drilling under magnification with ) 4. 3‑Dimensional (3D) animation based videos (n = 72) addressing: basic neurosurgery instruments,patient positions for surgery, Basic and complex surgical approaches 5. Social networking and blogs : discussion forum for addressing queries 6. Tele‑education: for neurosurgeons in India and Germany of a real classroom interaction in a cost-effective way | Question and answer Forum for open discussion on case based clinical scenarios and discussion of Interesting cases | 5226 visitors accessed the platform with 57% of repeat visitors. There were 64,380 views from 190 subscribers for surgical videos, 3‑D animation, graphics‑based training modules. The discussion forum had 968 members from 45 nations. 88.7% of the users used desktops and laptops as primary tool. About 10% of the visitors used tablets and cell phones for accessing the contents. | Not obtained | Not obtained | 1. Utilisation by mainly developed countries . Poor infrastructure, lack of interest in the governing bodies, irregular internet access, and language and cultural barriers could e the reasons for poor usage in developing countries 2. Content needs to be validated, structured and organised 3. Lack of peer review 4. Lite versions for use with weaker band width 5. Requires huge finances for establishment and maintenance of learning paltforms | 1. Can supplement training and education to acquire skills 2. Helps learn latest technologies and technique 3. The content on such platforms keep getting updated and will not suffer from stagnation | 1. Plan to Incorporate self evaluation modules such as ‘Computer Based Tests (CBT)’ and ‘Internet Based Tests (iBT)’, 2. Provision of synchronous learning by organizing webinars, assignments and online certifications with free access to all trainees across the globe. |  |
| 2 | Back A et al | 1. Lecture notes or scripts as PowerPoint (Microsoft PowerPoint, Microsoft Corp., USA) and PDF (Adobe System Inc., USA) scripts. 2. Podcasts were produced with Camtasia Studio (Version 5.1.0, TechSmith Corp., USA). 3. Virtual patient cases were provided by the CAMPUS authoring system (Version 1.3.2827 © 2006, University of Heidelberg, Germany). 4. Discussion forums or quiz formats were part of the LMS Blackboard features. 5. “WikiBlog” was based on the Team collaboration software Confluence (Atlassian, Australia) and the blogs by the open source web software WordPress MU (Free Software Foundation, USA). | Discussion forums and quizzes | During the semester, 38.6% of all students used the LMS daily, 48.3% on a weekly basis, and 13.1% less than once a week. | Feedback from faculty not obtained | Feedback was obtained from students using Survey monkey.  The lecture slides were used by most of the students (77.7%), followed by videos (71.9%) and digital texts (71.3%). The least used tools were simulations (33.4%), serious games (13.8%) and discussion forums (7%)  lThe students used LMS to gain study information (68.3%), for the preparation of exams (63.3%) and as preparation and post-processing of lessons (54.5%). The LMS was used for communicating with other students by 2.2% or even with teachers by 1.9% or for keeping lists or calendars (1.3%). | Faculty need to put more effort to add correct content  The tool should be interactive and provide face to face interaction | The LMS supports an learning and practice-oriented contents, which are easy to use. | 1. incorporating new features of the Web 2.0 and other means of teaching to attract students´ attention. 2. Use of proven tools might be more effective for a medical school and its teachers who aim to provide a good and satisfying education. 3. Retention of access to earlier learning materials. |  |
| 3 | Johnson CE et al | Ten common diagnoses were chosen for the educational content of PAP. Each module had objectives, reading material, pre test and evaluation.  The readings for modules were obtained from journals and textbooks | Pre and post test were conducted . Pre test had 4-7 questions while the post test had 8-14 questions with 2-5 repeats from the pre test . They were created using a software called Respondus | There were 7783 hits to PAP during the 2001–02 academic year. The mean number of hits to the PAP main page for all 80 residents was 95 (SD 56, range 0–222). A total of 1196 hits were made specifically to the required readings in the required modules. Hits to readings in the otitis media, asthma, gastroenteritis and fever modules totalled 440 (36.7%), 296 (24.8%), 238 (19.9%) and 222 (18.6%), respectively. WebCT counts hits to other tools in the course such as the objectives, pre- and post-tests, links to other resources and self-assessment tools. | Not obtained | The residents agreed that all the  module components were beneficial and that the  content had applications to patient care. | 1. The proficiency level that residents must achieve on test items in each module was not set, however WebCT can be easily programmed to set proficiency levels 2. Requires lot of time for preparing on-line course material 3. Need to be familiar with softwares and technology 4. Faculty development programs 5. Adequate computers 6. Relevance of online training 7. Time away from research | WebCT provides immediate feedback about performance on pre- and post-test items. Feedback is not only given in the form of a numerical score, but also as information and evidence about the best choice for the test item. This feature encourages self learning and assists residents in developing the lifelong learning skills during residency training.  LMS can support education about new clinical information and measure the knowledge acquisition of new clinical information  It helps to document participation and performance | Not mentioned |  |
| 4 | Halbert C | Twenty-nine,visual aids one page each and lecture summaries were posted to the content management system along with the relevant lecture presentations.  There were 22 handdrawn, one-page flow charts to summarize the biochemical pathways and bacterial classification schemes, and seven concise tables of cytokines and various disease families. . | Not mentioned about the assessments conducted | Mean individual student access to the online materials during the course was 6.3 times with a range of 0–42. A total of 82% of enrolled students accessed the online learning materials at least once during the course, 66% accessed the online materials two or more times | Not obtained | The majority (90%) reported that the online materials ‘‘sometimes’’ ‘‘often’’ or ‘‘always’’ positively affected their course preparation. Out of the students who utilized the online materials, 57% responded that the online material ‘‘rarely’’ or ‘‘never’’ replaced the course materials. The remainder (43%) responded that the online material replaced the course material ‘‘sometimes,’’ ‘‘often,’’ or ‘‘always.’’ | Not mentioned | There was a better understanding of the course material. | Not mentioned | this study  gave participants the option to utilize the online materials in  addition to traditional lectures and textbook readings |
| 5 | Herbert C | Recorded lectures  Powerpoints for each module . there were 29 such modules.  Each topic concluded with an interactive large group formative assessment session which focussed on integration of the topic content. | interactive questions and review quizzes using multiple question types with feedback  the module was not marked complete within the LMS until students obtained a score of 80% or more | 74% of enrolled students (n = 264) had attempted all 29 online modules and  85% of enrolled students had completed 90% or more of  the modules.  usage of the online modules was determined by data obtained from the SCORM packages | Not obtained | There was high overall student satisfaction with the online modules. There was a significant increase in student’s self reported rating of their understanding of topics after using the online modules.  Students commented that the modules enabled them to identify their overall study plan, and helped them to identify the areas where they most needed to focus their effort | -student time management was a challenge  -sequencing of module delivery in relation to other components | the modules proved to be meaningful and engaging and worked synergistically to provide a holistic experience that was highly effective in enabling and reinforcing student learning. | Not mentioned |  |
| 6 | Lebeaux D et al | Online teaching environments were constructed for the infectious diseases and microbiology course. The teaching materials included links to the national syllabus and videos for the topics.  Interactive quizzes during the lecture or at the end of the lecture followed by discussion of answers | Continuous assessments were conducted using quizzes | All students made at least one hit with a mean number of 200 ± 146 per student. Highest hits were recorded during the continuous assessment sessions | A satisfaction questionnaire regarding the perceived impact, benefits and limitations of Moodle was provided to the teache. Most of the teachers considered that this platform helped in teaching. All teachers planned to continue using Moodle over the subsequent academic years. | The students provided feedback regarding technical or educational aspects.  They suggested reducing the  time given to answer questions to reduce the risk of cheating and  to increase the time allotted for feedback. They also requested a 1-  week time interval between a lecture and the question related to it. | Assessment of introducing Moodle on student performance during the final exam could not be done – assessment of retention of knowledge could not be done  Reluctance of teachers to use online tools | The course management system attracted the interest of the students. The wide range of teaching possibilities, use of interactive activities and greater circulation of teaching materials | Not mentioned |  |
| 7 | Gaupp R | There were two modules – team work and error management. Video case studies were used .  Journal articles, videos, interactive quizzes, and podcasts were used to provide patient safety knowledge | -Online quizzes. --Reflections on real cases presented to them as a report or short video.  -critical peer feedback on their recensions of a scientific paper on patient safety. | 193 students participated |  | The students rated the online course as “average” (M= 2.93, SD = 0.8) on the 5-point Likert scale | The motivation of the students to use these platforms was not measured  The results cannot be generalised as response rate was poor. Since it was voluntary study, motivated students only participated. | The students with high levels of reflection or critical reflection reported higher self-regulated learning behavior. The e-learning system used facilitated self-regulated learning and metacognitive strategies by providing immediate feedback (and allowing discussions with peers. Hence, it supported those students who were unused to self-regulate their learning process independently. For students unable or unwilling to use cognitive and metacognitive strategies to improve their self-regulation, such online courses will have limited effects on either acquiring knowledge or develop beneficial attitudes towards patient safety. | Not mentioned |  |
| 8 | Chu A et al | A platform where students can document and share learning experiences that occur during clinical work. | A three-member faculty  panel reviewed the “learning moments.  The experienced clinical faculty led monthly in-person “Learning Moment Reflection” small group discussions to discuss and expand upon the ”learning moments” logged during their rotation | Within the first six months after implementation, 42 out of  53 (79.2%) students who rotated in our EM clerkship logged  at least one “learning moment” for a total of 323 “learning  moments” logged. Students have logged more than 1000 “learning  moments”  it did not affect their evaluation | Not obtained | Feedback collected in SUS questionnaires and interviews | Response rate was less  Findings cannot be generalised  that all e-learning tools will be the same | Simplicity and easy to use platform  It was compatible with their learning preferences | Not mentioned |  |
| 9 | Thepwongsa I et al | 20-hour human behaviour  course on KKUMEDX, in which this 18-topic course formed parts of  the human development and behaviour module. In this course, students  had twenty 1-h asynchronous online learning sessions and two 1-h sessions  for class discussion with course lecturers. The total numbers of  videos were 77 and the length of each video ranged from 0.22-49.01 min.  The total time of the videos for each topic ranged from 8.48-70.33 min. | After they completed the course, they had a multiple-choice test.  Although the scores were not the final grade for this module, the good  score level for the course was set at 56% and above. This cut point was  made based on the minimum passing level score of the module.  After the course finished, online questionnaires on Google Forms were  sent to the students in August 2020  The interview questions were developed based on the literature review,  findings of the survey mentioned above, and data from the LMS  monitoring system. The interviews were conducted online, in January  2021, and recorded through Google Meet, together with a writing record  by the interviewer (IT). Each interview took approximately 10 min. All  audio recordings were transferred to a desktop computer. Each interview  was listened to many times, then transcribed and saved as a Microsoft  Word document. The transcripts were then sent to each interviewee to  check for accuracy and to seek clarification on unclear comments. The  interview data were analysed manually. The first author (IT) coded  interview data directly on the printed transcripts. Each interview transcript  was read many times. Data were coded by only one coder. The  codes were then grouped into themes. Interrelating themes and  abstracting to smaller sets of themes were performed. | students completed the learning topics  ranging from 0-18 topics, mean 8.77 (SD 5.28). The total learning time of  the students ranged from 0-6848 min, and a mean of 908.13 (SD 933.24)  minutes. In terms of students' learning behaviour, 134 (47.35%) students  spent _10 h on the course and they often logged in to the course during  the morning (2780 logins, 45.3%) and evening hours (1678 logins,  27.3%). Although most of the students had good scores for this course (N  ¼ 249, 88.6%), there was no association between the good score and the  course attendance (COR ¼ 0.84; 95% CI ¼ 0.40–1.75), time spent in the  course (COR ¼ 1.55; 95% CI ¼ 0.61–3.91), or frequency of the course's  logins (COR ¼ 1.51; 95% CI ¼ 0.73–3.13). | Not obtained | students were satisfied and accepted this method  of learning, half of them still preferred in-class study  some students who had good scores but rarely or never  attended the course accepted that they could not understand the contents  by themselves. They, therefore, used other learning methods such as reading the summary notes from their peers or seniors.  the time spent in the course did not relate to the test score  may be due to the students’ learning styles.  students reported one of  the features of the LMS that they most preferred was the speed-up video,  in which they could adjust the speed as they wished. | 1. During the use of the LMS, the majority of medical students did not  recognise the barriers in online learning identified by previous studies  2. despite efforts to ensure  generalisability regarding the study population, this LMS had just been  launched for the first-year students which is the pre-clinical year and the  curriculum focused more on the basic medical sciences. Therefore, their  satisfaction and acceptance of the LMS may be different to the clinical  year students  3. this study was a single-institution study.  Considerably more work will need to be done across medical schools to  determine the effectiveness of this LMS on medical students in both preclinical  and clinical years to ensure generalisability  4. there is a potential for selection bias due to the  borderline participation rate of the study, the lack of randomisation.  5. as a result of using online questionnaires, the participants in  this study may be more familiar with online technology and have a  different characteristic background to the general population. Therefore,  it is important to bear in mind the possible bias in these responses | The results of  this study found that the usefulness, infrastructure, and user-friendliness  of the LMS played important roles in medical students’ online learning  experience (as shown in Table 3)  The results of our analysis indicated that the quality of  educational contents and perceived usefulness were found to be  marginally statistically significantly associated with the acceptance of  online learning on this LMS (as shown in Table 4). | need for further large-scale studies to ensure  generalisability. | Author contribution statement  Funding statement  Data availability statement  Declaration of interests statement  Additional information  Acknowledgements |
| **10** | **Seluakumaran K et al** | We designed  DPhysiol content using the following topic outlines.  *Interactive.* This section allowed communication with fellow students  and tutors through a discussion forum and an online chat room  (DPhysiol also allowed students to send personal messages to each  other or to tutors for private discussion). Students were encouraged to  post any doubts about the topics being taught, and both the students as  well as the tutors could reply to the questions posted. The contents of  all posts and replies were sent as e-mails to the corresponding students  and tutors. Another forum was created to allow tutors to post announcements  either to a specific group or to the entire class.  *Course information.* Information about the MBBS course structure,  contact details of staff and faculty members, and the various facilities  available for the students in our faculty were uploaded here. Students  were also provided with semester dates, a course timetable, and  physiology lecture contents and outcomes for the entire academic  year.  *Lecture notes.* The notes used by the lecturers during their traditional  style lectures were made available for download (in .pdf  format), usually immediately after the scheduled lectures. Throughout  the course, a total of 42 lecture files was uploaded.  *Laboratory exercises.* Students were involved in a total of 11  laboratory practical sessions. Laboratory exercises for each session  were uploaded before the classes so that students would be able to  read and prepare for the sessions.  *Problem-solving sessions.* The physiology teaching also consisted  of eight problem-solving sessions in which students were provided  with a set of problems that would be discussed with their respective  tutors in a tutorial setting. Questions for the problem-solving sessions  were uploaded before the scheduled sessions and regular announcements  were sent out to the students, requesting them to prepare the  answers before attending the sessions.  *Audiovisual resources.* A total of 24 Audiovisual (AV) resources in  the form of animations (.swf format) and videos (.mpeg format) that  were deemed relevant to the subjects being taught were uploaded by  the tutors. Students were able to either download or view them online.  For materials obtained from the internet, the relevant web sources  were acknowledged.  *Quizzes.* Seventeen quizzes, comprising various types of questions  (multiple choice, true-false, matching, filling in the blanks), were  designed and uploaded by tutors to test the level of understanding of  students about the concepts taught. Students were given appropriate time limits to complete each quiz. Only one attempt per quiz was  allowed, but students were able to review their attempts throughout  the academic year. The quiz settings also allowed students to receive  the correct answers, their marks, and feedback for the answers  immediately after their attempt. However, quiz marks were not included  in the formal assessment.  *Useful links.* Links to relevant sites such as the webpages of our  department, faculty, library, and the university’s student e-mail portal  were given. | *Online activity logs*  *Exam scores*  For the purpose  of this study, the physiology component marks in *part A* and *part*  *B* exams were compared with the activity logs of individual students  obtained from DPhysiol during *terms 1* and *2*, respectively. To assess  the Moodle usage between high and low achievers, we also compared  the activity logs of students who were in the top 10% of the overall  final physiology results (*part A* and *part B* exams combined) with  those of students who were in the bottom 10%.  We also assessed student participation and performance in online  quizzes and correlated these with their final exam marks  Finally, to evaluate the value of Moodle in improving student exam  performance, mean final physiology marks obtained by the students  (academic session 2008/2009) were compared with marks obtained by  the previous class (academic session 2007/2008), which did not use  the CMS  *Questionnaire.* During the 34th week of their course, a set of  questionnaires was handed to each of the students during tutorial  sessions to evaluate their feedback on the implementation of  DPhysiol. These anonymously answered questionnaires were in paper  format to include nonusers as respondents. The questionnaire consisted  of three parts. The first part provided the demographic information  (age, sex, and DPhysiol usage). The second part enabled  students to evaluate various elements of DPhysiol, including its  accessibility, ease of use, content, ability to facilitate student-student/  student-tutor interactions, and effectiveness in complementing faceto-  face teaching using a five-point Likert scale. The third part was a  narrative response section with four open-ended questions related to  students’ overall experience with DPhysiol. Nonusers were requested to respond to the first and third sections of the questionnaire to  evaluate their reasons for not using DPhysiol. | About 90% (*n* _ 194, 86 men and 108 women) of  first-year MBBS students registered as DPhysiol users. A total of 178 students (72 men and  106 women) responded to the questionnaire; 97.8% (*n* _  174) of them were DPhysiol users, whereas the remaining  2.2% (2 men and 2 women) were nonusers  Of the registered DPhysiol users, 90.7% of the students  (*n* _ 176) enrolled within the first 2 wk after DPhysiol was  made available. The site recorded a total of 6,347 visits and  51,935 hits during the entire academic session (*weeks 1–42*, a  total of 291 days).  Although there were no  significant differences between the average numbers of visits  per week for *term 1* (161 _ 57, mean _ SD) and *term 2*  (140 _ 55), the average number of hits per week was significantly  lower in *term 2* (875 _ 416) compared with *term 1*  (1,564 _ 811, *P* _ 0.05).  Students tended to use the site more frequently during their  study period compared with during the weeks of holiday and  exams. The mean number of hits on weekdays (204 _ 160  hits/day) was significantly higher than that of weekends (141 _  108 hits/day, *P* _ 0.05) during the study period.  When DPhysiol usage was compared by sex, mean hits per  day per student for male (0.98 _ 0.81) and female (0.84 _  0.65) DPhysiol users did not significantly differ.  The total hits for various resources made available in  DPhysiol are shown in Fig. 3. The most frequently used  content was the interactive functions (8,867 hits), which includes  a discussion forum (5,979 hits), announcement forum  (2,766 hits), and chat function (122 hits). Other frequently used  resources were lecture notes (8,075 hits), quizzes (4,006 hits),  and AV resources (2,580 hits). The average access per uploaded  lecture note was 192 hits/file, whereas accesses for AV  resources were 107 hits/file. Of all the quizzes available to  students, the most-attempted quiz recorded an attempt rate of  85.6% (166 of 194 users), whereas the least-attempted quiz  recorded only a 13.9% attempt rate (27 of 194 users).  The discussion forum had a total of 54 posts (52 by students  and 2 by tutors) and 121 replies (105 from students and 16  from tutors). A typical example of a forum discussion involving  student-student communication is shown in Fig. 4. For the  announcement forum, there were 35 postings by tutors.  There was a weak but significant correlation between students’  participation in the online quizzes and their final physiology  exam marks (Fig. 5A). A significant correlation was  also found between students’ marks obtained from the online  quizzes and their final exam marks (Fig. 5*B*).  When mean final physiology marks obtained by students in  the year of 2008/2009 were compared with the previous cohort  (2007/2008), there was a significant improvement in scores  (Table 2). However, no significant difference was detected in  the marks of the anatomy component between the two academic  years. | Not obtained | Most respondents commented  positively (strongly agree/agree) on the convenience of  the registration process (71.8%), accessibility (67.8%), and  ease of use (78.2%; Table 3, *statements 1–3*). Most of the users  (70.1%) were happy and satisfied to use DPhysiol (Table 3,  *statement 4*).  According to 85.1% of the respondents, the materials available  in DPhysiol were relevant and appropriate, and 88.5% of  them agreed that the materials were useful. When asked about  the use of DPhysiol as a communication tool, 56.9% of the  respondents believed that it allowed interactions with other  students (*statement 8*), whereas 55.1% agreed it enabled student-lecturer interactions (*statement 9*). Most respondents expressed  neutral (31.4%) to negative (37.7%) opinions on the  question regarding the potential of DPhysiol to replace traditional  learning (*statement 12*). Despite agreeing that DPhysiol  complemented traditional face-to-face learning (59.8%), students  still preferred to learn through conventional lessons,  which was evident from their responses to *statement 13*.  Respondents (78.1%) strongly recommended the continuation  of DPhysiol (*statement 14*), and 82.2% of them proposed that  a similar e-learning tool be used by other departments in the  medical faculty (*statement 15*). | 1. there was a lack of comprehensive, exam-based questions in the quiz section.  2. we noted that having lecture notes  online encouraged some students to use the CMS merely for  downloading them rather than using them as a learning tool.  3. the designing of the interactive functions was not attractive for students as no incentives were offered for their  Participation  4. Throughout the course, tutors only uploaded lecture notes  onto DPhysiol after their lectures, mainly due to the assumption  that students may decide to access the notes via the CMS  and skip lectures.  5. students also voiced concern over the poor internet  connection and download speed in the campus. | 1. CMS provides  an opportunity for cooperative learning.  2. The features available in the CMS promote interactions with learning resources  that can enhance student interest and motivation  3. The CMS allows students to decide where and  when they want to engage in learning, and it can have the effect  of bringing educators and students closer together  4. It was  found that students were willing to use DPhysiol outside  formal teaching hours, including on weekends and holidays.  This is a great advantage in courses such as medicine, where  students and educators are equally busy and have limited  physical interactions with one another | Not mentioned | Declaration of interests statement  Acknowledgements |
| **11** | **Kolcu G et al** | Via  the learning management system, the curriculum and  resources are shared with students, debate forums for  students are provided, homework and task management  are made, feedback is received from the students  and lecturers, and assessment and evaluation are made  accordingly.  In evaluating the elements belonging to the LMS  used by the students, it was seen that the system was  primarily used for course videos, learning resources  apart from course videos, and assessment and evaluation | In our medical school 1023 learning sources, 589 live  lectures were made and total 345 hours of video records  taken between 17.04.2020 and 09.05.2020 with  1738 students, 198 lecturers. After the live lessons, the  video was transferred to the learning management system  and made accessible for students.  Our faculty started the online assessment and  evaluation process with the neurosurgery and emergency  medicine internship exams on 15/04/2020, and  subsequently carried out a total of 40 measurement/  evaluation applications including 37 shelf exams, 5 internship  exams, and 3 preclinical board exams within  one month. | - | Not obtained | In the study, the LMSAS developed by Sezer was  used (11). The LMSAS consists of 21 questions. The  scale consists of four subdimensions, the performance  expectancy (PE) represents the expectation of an increase  in performance with the use of technology; effort  expectancy (EE) represents the belief that the use  of technology will be easy; facilitating conditions (FC)  represents the conviction that there are several supportive  elements in the use of technology,and social  influence (SI) represents the opinions of the important  persons in the environment where the mentioned  technology shall be used. The scale has 8 questions for  performance expectancy (question 1, 2, 3, 4, 5, 6, 7,  and 8), 5 questions for effort expectancy (question 9, 10, 11, 12, and 13), 5 questions for facilitating conditions  (question 14, 15, 16, 17, and 18), and 3 questions  for social influence (question 19, 20, and 21). The  scale was answered using a Likert scale. The names of  the options in the scale and their scores are: totally  disagree (1 point), disagree (2 points), undecided (3  points), agree (4 points), and totally agree (5 points).  The reverse scoring method is not used in the scale.  The minimum and maximum scores that can be obtained  from the scale are 21 and 105, respectively. High  scores indicate a high level of acceptance of LMS by  the students.  For evaluating satisfaction “Anadolum eCampus  System Satisfaction Questionnaire” preferred which  was used in Anadolu University Open Education System  (17). The questionnaire consists of two parts. The  first part questions are; “To which extent were you  satisfied with the system?”, “To which extent do you  think the system helped you learn?”, “How easy was  it for you to access the information and resources you  were looking for in the system?”, “To which extent did  you find the support services offered about the system  useful?” and “To which extent was the system beneficial  in preparing you for the exam?”, The answers  structured in the form of a five-point Likert, where  the answers vary between “I am not satisfied” and “I  am very satisfied”. In the second part, opinions about  the positive and required aspects of LMS were taken.  In the qualitative component of the study, in the third  part, students were asked open-ended two questions  to express their views on the positive and aspects that  should be improved regarding distance education. Answers  coded under themes by researchers. | Although sharing  a situation specific to our faculty is a limitation of the  study, it suggest the evaluation of other faculties | 1.the strong intentions of  the clinical students regarding the use of LMS are  considered to be positive  2. The data and analyses are derived from  a cross-sectional design, so causal inferences are difficult  to draw.  3.Because formal education was  interrupted due to the pandemic, we used an online  data collection method in the study, which resulted in  the sampling of our study being voluntary. Therefore,  the possibility of selection bias should be considered.  4.Tthe fact that the study was applied  specifically to Suleyman Demirel University, Faculty  of Medicine is considered as a limitation of the study, | the reluctance of the nonclinical students to these systems should be considered  as an important area of research within the  framework of program evaluation. | Conflict of interest declared  Acknowledgement |
| 12 | Thepwongsa I et al | Given in supplementary material | Semi-structured phone interviews were conducted for the pilot testing of the LMS. Five family medicine residency trainees and eight family medicine academic staff members were invited to enroll in the online diabetes course and provide feedback on their online learning experience. Informed consent was obtained from all participants. The participants were asked to complete the course and then for their phone feedback. Data were collected from both the LMS monitoring system and phone interviews.  The newly developed online LMS was evaluated through two surveys:  First, a short survey was conducted to explore learners’ opinions about factors influencing adoption of online learning to gain learners’ perspectives and obtain data for customization of the LMS to serve learners’ need  Second, a satisfaction survey was conducted with 255 learners enrolled in 13 courses. These courses were taught solely online (no face-to-face sessions for these courses) through KKUMEDX. |  |  | Table 2 and table 4 in the article | 1. present study was a single-institution study.  2. the use of the LMS is still in a short period of approximately two more years.  3. potential for selection bias in observational studies owing to the lack of randomization  4. present study used online questionnaires, and the participants in this study may have a different characteristic background from the general population since they may be more familiar with online technology. | - | 1.further large-scale studies involving many medical schools are required to ensure generalizability  2. ongoing monitoring and observation of learners’ behavior in online learning are needed | **Acknowledgments**  **Funding**  **Data availability statement**  **Disclosure statement** |
| 13 | Dash S | The content shared in the Google  Classroom can be broadly classified into five types: (1) lecture  notes, which included the powerpoint presentation to  be used for the lecture classes and incomplete notes for  class activity; (2) quiz, comprising of MCQs, short answer  questions, multiple true and false; (3) learning resources,  including links to articles and YouTube videos explaining  concepts; (4) announcements about forthcoming activities in  the classroom; and (5) assignments requiring submission in  the Google Classroom platform | a feedback  form having nine questions in a Likert Scale format, prepared  using Google Forms, was posted in the Google Classroom. | The content shared in the Google  Classroom can be broadly classified into five types: (1) lecture  notes, which included the powerpoint presentation to  be used for the lecture classes and incomplete notes for  class activity; (2) quiz, comprising of MCQs, short answer  questions, multiple true and false; (3) learning resources,  including links to articles and YouTube videos explaining  concepts; (4) announcements about forthcoming activities in  the classroom; and (5) assignments | Not obtained | Of the 41 students who submitted the completed feedback  form were 61% females and 34.1% males. Two students  chose not to reveal their gender. A total of 80.5% students  found accessing class notes using Google Classroom easier.  Same percentage of students found YouTube videos posted  in the platform helpful. A total of 17.1% students were neutral  about it. Although 68.3% found it easy to answer “quiz”  on Google Classroom, 7.3% did not and 12.2% were neutral  about it. | 1.limitation of using handheld  device in writing and submitting complex material for  assessment, which e-learning tool developers need to  look into.  2. lack of testing for skills and attitude  3. dimensions  such as ease of navigation, fun or boring aspects, independence  and collaboration potential, need to be evaluated to  test for efficacy of LMS. | 1.fostering teaching  and learning outside of the classroom environment  2. Items inquiring about better  access to learning material, use of additional learning  resource like YouTube videos scored high indicating effectiveness  of this LMS  3. immediate feedback receiving  4. use of Google Classroom on mobile phones  was preferred over its use using laptops  5. Google Classroom provides  for an effective alternative to other costly products.  6. Google Classroom can assist in enhancing  faculty effectiveness and efficiency  7. Using it in our setup neither  imposed extra cost to students nor to the institution. No  additional cost for creation of e-learning resources was  incurred.  8. LMS can facilitate personalization  of learning, individualization of tracking, assessment, and  the following required support | 1.providing more data on usage by students like  viewing of resources, providing log files, ease of image  upload, more Microsoft Word processing tools, and so forth,  can make it more effective | - |
| 14 | Çakmakkaya 0S et al | The system included an overall course page, content page that included previously recorded lectures, slideshows and learning materials, an announcement page, and an assignment and exam page. Lectures were given as syn­chronous live streaming, and additionally, they were recorded to provide asynchronous learning options. Stu­dents were able to access the presentations and prepara­tion materials beforehand. All students were assigned a personal ID for access to the system. |  |  | Not mentioned | The questionnaire consisted of three different parts with 24 questions. The first part included demographic items (five questions). The second part consisted of eleven statements regarding teaching methods, techni­cal opportunities, and attitudes toward online education, which were rated on a 5-point Likert scale. The third part of the questionnaire consisted of open-ended questions, which aimed to determine the strengths, weaknesses, and developmental aspects of online learning (seven ques­tions). Additionally, we asked for the overall satisfaction score as the primary outcome of the study, which was measured on a 10-point scale  Positive association with students’ overall satisfaction   1. Being familiar with online teaching techniques and reporting good IT literacy. 2. Having their own personal workspace. 3. Satisfaction with faculty members’ online teaching skills. 4. Interactive teaching methods used by faculty members. 5. Self-reported longer attention span during online learning.   Negative association with students’ overall satisfaction   1. Encountering technical problems. 2. Believe that lecture hall lectures are more effective. | timing of the survey fell into the early months of the pandemic and reflects the experience with online learning when we were rushed to provide continuity of our educational program. the results might not be generalizable to post-pandemic well-organized and well-structured online learning activities | One of the positive gains of the pandemic for us was the establishment of a good Learning Management System and the development of organizational plans not only for future blended learning but also for possible emergencies such as new pandemics or natural disasters. | In the post-pandemic era, well-planned and developed blended learning programs will increase the students’ satisfaction and will help us to use students’ time more effectively. Our study suggests that using interactive teaching methods, pedagogically improved online teaching skills, adjusting the duration of lectures according to students’ attention span, and addressing the disparity of resources by supporting students on techni­cal and workplace issues might positively affect the stu­dents’ satisfaction with our online learning program. | Abbreviations  Acknowledgements  Author contribution  Funding  Data availability  Declaration  (Ethics approval, consent for publication, competing interrests |
| 15 | Gokli A et al | The LMS has 289 courses, organized into 18 subtopics, with  each course consisting of recorded lectures, multiple-choice  assessments and PDF reference articles available with a single  click (Fig. 4). A summary of courses and enrollment to date is  given in Fig. 5. Subspecialties are broken into clinical radiology  specialties (e.g., chest, fetal, gastrointestinal, neonatology) and academic/research-related topics (e.g., center  for contrast ultrasound, academic promotion, image quality,  technology). An automated program interface was developed  to connect to our customized teaching file in the  Primordial-Nuance (Nuance Communications, Burlington,  MA) workflow manager, adding more than 500 teaching cases  in all subspecialties to RADIAL  toolbox tile - short (15-min) department-made lectures,  highly cited articles, department-created PDF tutorials,  figures/tables from seminal articles or lectures, report templates  and measurement guides  Ultrasound checklist app - | - | - | - | - | The Information Services Team has changed its browser preference  several times since RADIAL was introduced; with this  there has been a change in the “single sign-on” option, which  has been confusing for some users.  onboarding process has not been consistent for all of our frequently  registered new users, meaning that some find they do  not have access to all materials as promised and do not know  how to fix these issues  In reality, busy radiologists and trainees are reluctant  to spend time learning a new technology despite knowing  that it will likely save time and improve learning |  | This type of LMS has the potential to be applied to other  radiology departments. To assess scalability, a separate project  is underway to include five outside pediatric institutions in  the testing and evaluation of the system. The end goal for the  next phase of RADIAL will be to replace competency with  subject mastery by providing point-of-care information and  encouraging deliberate practice in trainees and attending radiologists | Compliance with ethical standards  Conflict of interest |
